# Lipid Messenger Phosphatidylinositol-4,5-Bisphosphate Is Increased by Both PPARα Activators and Inhibitors: Relevance for Intestinal Cell Differentiation

**DOI:** 10.3390/biology11070997

**Published:** 2022-06-30

**Authors:** Katerina Cizkova, Katerina Koubova, Zdenek Tauber

**Affiliations:** Department of Histology and Embryology, Faculty of Medicine and Dentistry, Palacky University, 779 00 Olomouc, Czech Republic; katerina.cizkova@upol.cz (K.C.); katerina.koubova@upol.cz (K.K.)

**Keywords:** fibrates, intestinal cell differentiation, phosphatidylinositol-4,5-bisphosphate, carcinogenesis

## Abstract

**Simple Summary:**

Fibrates, such as fenofibrate, are widely used drugs for dyslipidaemia treatment. It is known that they activate peroxisome proliferator-activated receptor α (PPARα) which serves as a lipid sensor in the organism. This article addresses how activators and inhibitor of the PPARα could affect differentiation of intestinal cells. Carcinogenesis is a disruption of normal differentiation process and colorectal carcinoma is the third most common cancer in terms of incidence, but the secondp in terms of mortality. One of the important signalling pathways in intestinal cell differentiation as well as carcinogenesis is PI3K/Akt/PTEN. We showed that PPARα activators as well as inhibitor affected the levels of one member of this pathway called phosphatidylinositol-4,5-bisphosphate. This molecule is important for formation of microvilli, the essential structures of fully differentiated intestinal cells.

**Abstract:**

We investigated the effects of PPARα activators fenofibrate and WY-14643 as well as the PPARα inhibitor GW6471 on the PI3K/Akt/PTEN pathway of intestinal cell differentiation. Our previous study showed that all these compounds increased the expression of villin, a specific marker of intestinal cell differentiation in HT-29 and Caco2 cells. Our current results confirmed the central role of lipid messenger phosphatidylinositol-4,5-bisphosphate (PIP2), a known player in brush border formation, in mediating the effects of tested PPARα ligands. Although all tested compounds increased its levels, surprisingly, each of them affected different PIP2-metabolizing enzymes, especially the levels of PIP5K1C and PTEN. Moreover, we found a positive relationship between the expression of PPARα itself and PIP2 as well as PIP5K1C. By contrast, PPARα was negatively correlated with PTEN. However, the expression of antigens of interest was independent of PPARα subcellular localization, suggesting that it is not directly involved in their regulation. In colorectal carcinoma tissues we found a decrease in PTEN expression, which was accompanied by a change in its subcellular localization. This change was also observed for the regulatory subunit of PI3K. Taken together, our data revealed that fenofibrate, WY-14643, and GW6471 affected different members of the PI3K/Akt/PTEN pathway. However, these effects were PPARα-independent.

## 1. Introduction

Peroxisome proliferator-activated receptor α (PPARα) is a ligand-dependent transcription factor which is involved in regulation of various cellular functions, including energy metabolism, oxidative stress, immune response, xenobiotic metabolism, cell proliferation, differentiation, and carcinogenesis [1,2,3,4,5]. PPARα can be found in both the cytoplasm and nucleus, dynamically shuttling between these two compartments. Ligand binding favors nuclear localization of the receptor, its heterodimerization with the retinoid X receptor, and recognition of peroxisome proliferator response elements (PPREs) in the promotor of target genes [3,6]. Fibrates, such as fenofibrate, bezafibrate, clofibrate, and gemfibrozil, are among the most important ligands of PPARα. For decades, fibrates have been widely used in clinical practice for the management of hypertriglyceridemia, with relatively good safety profiles [7,8,9]. In addition to fibrates, a variety of endogenous and exogenous compounds such as dietary fatty acids, eicosanoids, phthalates, and pesticides also serve as PPARα ligands [1,2]. Moreover, PPARα actions could also be modulated in a ligand-independent manner via phosphorylation [10]. PPARα is not the only member of the PPAR receptor family. Other isoforms, PPARβ/δ and PPARγ, with various biological functions, have been described [1,3].

Although it has been reported that PPARα plays a role in the differentiation of various cells [11,12,13,14,15,16,17], the differentiation of intestinal cells seems to be PPARα-independent, as shown in our previous study [18]. In HT-29 and Caco2 cells lines, administration of PPARα activators as well as a PPARα inhibitor increased the expression of villin. It is an epithelial-specific actin regulatory protein which is considered an intestinal differentiation marker because its expression is increased in differentiated cells. It is known that the association of villin with the lipid messenger phosphatidylinositol-4,5-bisphosphate (PIP2) enhances its actin bundling function and thus the formation of brush borders [19,20,21]. Based on these, one can speculate, that the effect observed for the PPARα activators as well as the PPARα inhibitor could be (at least partially) mediated via PI3K/Akt/PTEN signaling because it is affected by PPARα ligands in both PPARα-dependent as well as PPARα-independent manners [22,23,24,25,26]. The PI3K/Akt/PTEN pathway is one of the important players in intestinal cell differentiation as well as in colorectal carcinoma. Phosphatidylinositol-4-phosphate-5-kinase (PIP5K) synthesizes PIP2 which is phosphorylated by phosphatidylinositol-3 kinase (PI3K) to phosphatidylinositol-3,4,5-triphosphate (PIP3). The latter then activates Akt, which is downstream effector of PI3K [27]. The reaction catalyzed by PI3K is reversed by a phosphatase and tensin homolog (PTEN), which dephosphorylates PIP3 and converts it to PIP2 [28]. PI3K/Akt is an oncogenic pathway, and it is involved in the proliferation of intestinal epithelial cells in vitro and in vivo [29], while PTEN is a known tumor suppressor [28].

The aim of this study was to investigate whether the effect of two PPARα activators (fenofibrate and WY-14643) as well as a PPARα inhibitor (GW6471) on levels of PIP2-metabolizing enzymes, namely PIP5K1C, PI3K, and PTEN, as well as PIP2 itself, in HT-29 and Caco2 cells could explain the previously detected increase in villin expression [18]. Moreover, we investigated whether there could be a link between PPARα expression levels and the levels of the currently studied targets after PPARα ligand treatment. In addition, the immunohistochemical profiles of PIP5K1C, PIP2, PI3K, and PTEN in poorly differentiated colorectal carcinomas and adjacent normal colon tissue were examined and compared with PPARα levels.

## 2. Material and Methods

### 2.1. Cell Culture and Treatment

HT-29 and Caco2 cells (human colorectal carcinomas) were cultured and treated under the same conditions and experimental scheme as we described in our previous study [18]. The cell lines were originally obtained from American Type Culture Collection and authentication before experiment via STR profiles was performed by the Department of Clinical Genetics, Palacky University Olomouc. The cells were routinely cultured in DMEM (Sigma Aldrich, D6171) supplemented with 10% (HT-29) and 15% (Caco2) FBS (HyClone, SV30160.03), penicillin (100 U/mL), and streptomycin (100 mg/L)). Cells were incubated at 37 °C and 5% CO_2_ and passaged twice per week.

The stock solutions of fenofibrate (Cayman chemicals, Ann Arbor, MI, USA; cat. no. 10005368), WY-14643 (Sigma-Aldrich; cat. no. C7081), and GW6471 (Cayman chemicals, cat. no. 11697) were prepared by dissolving in DMSO. The cells were seeded in 96-well cultivation plates (TPP, cat. no. 92696) at density of 10,000 cells/well (HT-29) and 7000 cells/well (Caco2) for In-Cell ELISA and in 8-well cell culture slides (SPL Life Sciences, Pochon, Korea, cat. no. 30108) at density of 18,000 cells/well for immunofluorescent multiplex staining and adhered overnight. The next day, the cells were treated with fenofibrate, WY-14643, or GW6471 in following concentrations: 25 μM and 150 μM (HT-29) or 200 μM (Caco2) fenofibrate, 25 μM and 200 μM WY-14643, and 1 μM and 10 μM GW6471 and were incubated for 72 h at 37 °C. The control cells were treated with appropriate concentrations of DMSO. The used concentrations were determined using WST-1 proliferation test (Roche, cat. no. 11 644 807 001). The results for this assay under the same experimental conditions were published in our previous study [18] and can be found in supplementary material.

To obtain differentiated cells, HT-29 cells were seeded, incubated overnight and then treated with 5 mM sodium butyrate (Sigma Aldrich, St. Louis, MO, USA, cat. no. B5887) for 72 h. For obtaining differentiated Caco2 cells, the cells were cultured 14 days after reaching confluence. The growth medium was changed twice per week [30]. After that, the medium was changed and the cells were treated with PPARα ligands for 72 h as mentioned above. Differentiated cells used as controls were treated with appropriate concentrations of DMSO instead of PPARα ligands. The cells were not reseeded during the experiments.

### 2.2. In-Cell ELISA (ICE)

The changes in expression of proteins of interest and lipid messenger PIP2 were investigated by In-Cell ELISA colorimetric kit (Thermo Scientific, Waltham, MA, USA, cat. no. #62200). The procedure was performed according to the vendor’s protocol. After incubation period, the cells were washed in PBS and fixed with 4% paraformaldehyde for 10 min at RT. The following primary antibodies were used: PTEN (GeneTex, Hsinchu, Taiwan, cat. no. GTX83304) at dilution 1:2000, PIP5K1C (Invitrogen, #702444) at dilution 1:500, PI3K p85/p55 (Invitrogen, Waltham, MA, USA, cat. no. #710400) at dilution 1:100, PIP2 (Invitrogen, #MA3-500) at dilution 1:500. The incubation period took overnight at 4 °C. After that, the samples were incubated with horseradish conjugate, followed with TMB substrate (parts of the In-Cell ELISA colorimetric kit). The measured antibody signals (absorbance at 450 nm) were normalized to whole-cell staining using Janus Green (absorbance at 615 nm). The results are shown as relative expression [%] in comparison to appropriate control cells (100%). The absorbance was measured by microplate reader Power Wave XS (Bio-Tek, Winnoski, VT, USA). The experiment was performed in three independent duplicates (*n* = 6).

### 2.3. Multiplex Immunofluorescent Staining

To assess whether the changes in PIP5K1C, PIP2, and PTEN levels detected by In-Cell ELISA are dependent on subcellular localization of PPARα, we colocalized the PPARα with antigens of interest. The reason for this is that the first prerequisite for the regulation of gene expression is the nuclear localization of the receptor. Undifferentiated HT-29 cells were seeded in 8-well cell culture slides, adhered overnight, and treated with 150 μM fenofibrate or 10 μM GW6471 for 72 h. After that, the cells were fixed with 4% paraformaldehyde for 10 min at RT. For multiplex immunofluorescent staining, we used Opal™ 4-Color Manual IHC Kit (Akoya Biosciences Marlborough, MA, USA, cat. no. NEL810001KT) according to the vendor’s protocol. The following primary antibodies were used: PIP5K1C (Invitrogen, #702444) at dilution 1:100, PIP2 (Invitrogen, #MA3-500) at dilution 1:100, PTEN (GeneTex; GTX83304) at dilution 1:50, and PPARα (GeneTex; GTX28934) at dilution 1:100. The incubation period with primary antibodies took 1 h at RT.

The multiplex immunofluorescent staining was followed by image analysis using ImageJ software. The intensities of fluorescence of PIP5K1C, PTEN, and PIP2 were measured separately for cells with nuclear positivity of PPARα and for cells with PPARα negative nuclei (30 cells in each group from different fields of vision) to determine whether the immunofluorescent intensity is dependent on subcellular localization of PPARα.

### 2.4. Immunohistochemical Staining

Tissue samples of poorly differentiated (grade 3) colorectal adenocarcinoma and adjacent normal colon tissue from 8 patients (5 males, 3 females) were obtained from the archives of the Department of Clinical and Molecular Pathology, Faculty of Medicine and Dentistry, Palacky University Olomouc. The basic patient characteristics (age, sex, grading, TNM staging) are provided in Table 1. The use of all samples was approved by the Ethics Committee of the University Hospital and the Faculty of Medicine and Dentistry, Palacky University in Olomouc (protocol No. 134/14 dated 21 August 2014).

Antigens of interest were detected in 4 µm thick paraffin sections using following primary antibodies: PTEN (GeneTex; GTX83304) at dilution 1:1500, PIP5K1C (Invitrogen, #702444) at dilution 1:100, PI3K p85α (Santa Cruz, Dallas, TX, USA, ca. no. sc-1637) at dilution 1:50, PIP2 (Invitrogen, #MA3-500) at dilution 1:900. After deparaffinization and hydration, heat-induced antigen retrieval in citrate buffer pH6 was performed (120 °C, 15 min, Histos device). Then, the samples were pre-treated with PolyDetector Peroxidase Blocker (Bio SB, part of the detection kit) for 5 min and 30 min with ProteinBlock (Dako, Glostrup, Denmark). Then, the slides were incubated with primary antibodies for 1 h at RT and the reaction was visualized by Mouse/Rabbit PolyDetector DAB HRP Brown kit (Bio SB, Santa Barbara, CA, USA, cat. no. BSB 0205). Tris buffer with TWEEN 20 (pH 7.6) was used for washing between different steps. Nuclei were counterstained with hematoxylin. After washing in tap water, the samples were dehydrated and cover slipped. Before evaluation, the slides were coded to prevent observer bias. The slides were evaluated twice at different times. Evaluation of staining intensity was semiquantitative, performed as follows: 0 for negative tissue, 1 for weak signals, 2 for moderate signals, and 3 for strong signals. The expression of PPARα in the same patients was estimated in our previous study [18] with the same staining procedure.

### 2.5. Statistics

The results obtained from In-Cell ELISA were evaluated by one sample *t*-test to control value 100%. The intensity of fluorescence measured with ImageJ was evaluated using unpaired *t*-test. The results obtained from immunohistochemical staining of tissue samples were evaluated by Wilcox test. The correlation between relative expression of PIP5K1C, PIP2, PI3K, PTEN, and PPARα after PPARα ligand treatment were evaluated using Spearman correlation coefficients. All calculations were performed by Graph Pad Prism 8 at the *p* < 0.05 level of significance. Statistically significant differences are marked with asterisk (*) directly in graphs: * *p* ≤ 0.05, ** *p* ≤ 0.01, *** *p* ≤ 0.001, **** *p* ≤ 0.0001.

## 3. Results

### 3.1. Effect of Fenofibrate, WY-14643 and GW6471 on Levels of Lipid Messenger PIP2 in Colorectal Carcinoma HT-29 and Caco2 Cell Lines

**Figure 1:**
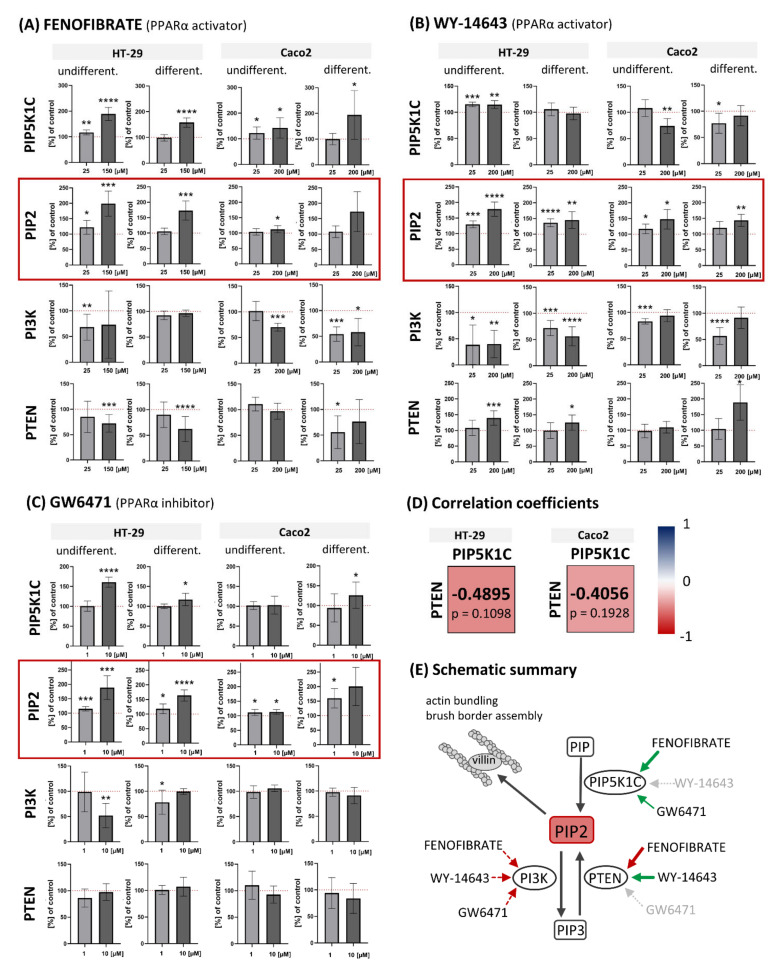
Effect of PPARα activators fenofibrate (**A**) and WY-14643 (**B**) and PPARα inhibitor GW6471 (**C**) on expression of PIP5K1C, PIP2, PI3K, and PTEN. Relative expression of proteins of interest in comparison to control was measured by In-Cell ELISA. Results are shown as mean ± SD (*n* = 6). Red dotted line represents control cells: DMSO treated undifferentiated or differentiated cells (100%). HT-29 cells were differentiated by sodium butyrate, Caco2 cells were differentiated by post-confluent growth (14 days). Note the increase in PIP2 expression in both used cell lines after PPARα ligands treatment (red rectangles). Statistically significant results in comparison to control cells are marked by * *p* ≤ 0.05, ** *p* ≤ 0.01, *** *p* ≤ 0.001, **** *p* ≤ 0.0001. (**D**). Relationship between changes in expression of PIP5K1C and PTEN in HT-29 and Caco2 cell lines are shown as Spearman correlation coefficients. Changes in expression of these proteins after PPARα ligand treatment showed a moderate negative correlation in both cell lines. (**E**) Schematic summary of obtained results. Detected increase in PIP2 observed after fenofibrate, WY-14643, and GW6471 treatment could explain our previous results reported increase in villin expression under the same experimental conditions [18]. The effect of PPARα ligands is illustrated by arrows: green—increase in expression, red—decrease in expression, grey dotted—no effect, red dotted—decrease in expression or no effect (the results differ in HT-29 and Caco2 cell lines).

All three tested PPARα ligands showed one common feature in both cell lines: concentration-dependent increase in levels of PIP2 (see Figure 1A–C). Generally, the effect of PPARα ligand treatment was more pronounced in HT-29 cells than in Caco2. In HT-29 cell line, 150 μM fenofibrate increased the PIP2 level to 199.0% of control (*p* = 0.0002) in undifferentiated cells and to 172.8% of control (*p* = 0.0003) in differentiated ones; 200 μM WY-14643 increased the PIP2 level to 178.6% of control (*p* < 0.0001) in undifferentiated cells and to 144.9% of control (*p* = 0.0022) in differentiated ones. Finally, 10 μM GW6471 increased the PIP2 level to 188.7% of control (*p* = 0.0005) in undifferentiated cells and to 163.9% of control (*p* < 0.0001) in differentiated ones. In the Caco2 cell line, 200 μM fenofibrate increased PIP2 level to 112.3% (*p* = 0.0198) in undifferentiated cells and to 172.9% (*p* = 0.1086) in differentiated ones; 200 μM WY-14643 increased the PIP2 level to 147.7% of control (*p* = 0.0129) in undifferentiated cells and to 143.8% of control (*p* = 0.0024) in differentiated ones. Finally, 10 μM GW6471 increased the PIP2 level to 113.3% of control (*p* = 0.0120) in undifferentiated cells and to 200.4% of control (*p* = 0.0553) in differentiated ones.

### 3.2. Effect of Fenofibrate, WY-14643 and GW6471 on Expression of PIP5K1C, PI3K (p85/p55) and PTEN in Colorectal Carcinoma HT-29 and Caco2 Cell Lines

Our results showed that the tested PPARα ligands regulate PIP2 levels by targeting different enzymes involved in its turnover, namely PIP5K1C, PI3K, and PTEN (see Figure 1). Higher concentrations of fenofibrate significantly increased the expression of PIP5K1C in both cell lines. In the HT-29 cell line, 150 μM fenofibrate increased it to 188.9% of control (*p* < 0.0001) in undifferentiated cells and to 157.8% of control (*p* < 0.0001) in differentiated ones. In the Caco2 cell line, 200 μM fenofibrate increased PIP5K1C expression to 142.5% of control (*p* = 0.0195) in undifferentiated cells and to 194.1% of control (*p* = 0.0397) in differentiated ones. Contrary to fenofibrate, the second tested fibrate molecule, WY-14643, did not cause distinct increase in PIP5K1C expression. In HT-29 cell lines, the obtained results were 114.8% of control (*p* = 0.0019) and 97.2% of control (*p* = 0.6395) for undifferentiated and differentiated cells, respectively. In Caco2 cells, 200 μM WY-14643 treatment decreased PIP5K1C expression to 74.0% of control (*p* = 0.0012) and to 91.5% of control (*p* = 0.3293) in undifferentiated and differentiated ones, respectively. Administration of PPARα inhibitor GW6471 also led to increase in PIP5K1C expression as in the case of fenofibrate but to a lesser extent. In HT-29 cells, 10 μM GW6471 increased PIP5K1C expression to 160.9% of control (*p* < 0.0001) in undifferentiated cells and to 117.1% of control (*p* = 0.0274) in differentiated ones. In Caco2 cells, we detected PIP5K1C levels of 103.1% of control (*p* = 0.7084) in undifferentiated cells and of 125.9% of control (*p* = 0.0153) in differentiated cells after the administration of 10 μM GW6471.

The expression levels of PI3K were decreased or unaffected after PPARα ligand treatment in comparison to control cells. In HT-29 cells, 200 μM WY-14643 led to decreases in PI3K expression in both undifferentiated as well as differentiated cells (39.9% of control (*p* = 0.0067) and 56.4% of control (*p* < 0.0001), respectively), whereas 150 μM fenofibrate and 10 μM GW6471 led to a decrease in PI3K only in undifferentiated cells (73.1% of control (*p* = 0.4724) and 51.9% of control (*p* = 0.0018), respectively). There were no effects of 150 μM fenofibrate and 10 μM GW6471 on differentiated HT-29 cells (96.4% of control (*p* = 0.3359) and 99.2% of control (*p* = 0.9257), respectively). In Caco2, 200 μM fenofibrate decreased PI3K expression in both undifferentiated as well as differentiated cells (70.1% of control (*p* = 0.0002) and 58.0% of control (*p* = 0.0119), respectively). Contrary to HT-29 cells, 200 μM WY-14643 had no effect on PI3K expression in undifferentiated as well as differentiated Caco2 cells (93.9% of control (*p* = 0.2498) and 97.7% of control (*p* = 0.5388), respectively). Surprisingly, a lower concentration (25 μM) of WY-14643 significantly decreased PI3K expression (83.1% of control (*p* = 0.0005) and 56.5% of control (*p* < 0.0001), respectively). GW6471 had no effect on PI3K expression in Caco2 (105.9% of control (*p* = 0.1683) and 91.1% of control (*p* = 0.3501), respectively).

Surprisingly, higher concentrations of fenofibrate decreased the expression of PTEN in both cell lines. In the HT-29 cell line, 150 μM fenofibrate decreased it to 72.1% of control (*p* = 0.0002) in undifferentiated cells and to 62,3% of control (*p* < 0.0001) in differentiated ones. In the Caco2 cell line, 200 μM fenofibrate led to PTEN expression of 97.0% of control (*p* = 0.7242) in undifferentiated cells and to 76.45% of control (*p* = 0.3518) in differentiated ones. Contrary to fenofibrate, the administration of WY-14643, another PPARα activator, caused an increase in PTEN expression. In HT-29 cell lines, the obtained results were 139.7% of control (*p* = 0.0018) and 125.4% of control (*p* = 0.0207) for undifferentiated and differentiated cells, respectively. In Caco2 cells, 200 μM WY-14643 treatment led to relative expression of PTEN enzyme of 109.7% of control (*p* = 0.2564) and 188.8% of control (*p* = 0.0245) in undifferentiated and differentiated ones, respectively. Administration of PPARα inhibitor GW6471 had no effect on PTEN expression in either HT-29 or Caco2. PTEN levels reached 97,6% of control (*p* = 0.6988) and 107.3% of control (*p* = 0.2831) in undifferentiated and differentiated HT-29 cells, respectively. In Caco2, the relative expression of PTEN was 92.7% of control (*p* = 0.3167) and 83.93% of control (*p* = 0.2716) in undifferentiated and differentiated cells, respectively.

Interestingly, the tested PPARα ligands revealed the opposite effect for expression of PIP5K1C and PTEN. We found a moderate negative correlation with Spearman r = −0.4157 (*p* = 0.0434, *n* = 24). The effect was very similar in both cell lines. If we evaluated the HT-29 and Caco2 cells separately, the obtained Spearman correlation coefficients reached similar values, specifically −0.4895 for HT-29 and −0.4056 for Caco2 (*n* = 12 for each cell line) (Figure 1, part D). We found negligible or no relationship between the expression of PI3K and PIP5K1C (r = −0.1435, *p* = 0.5036) and PI3K and PTEN (r = −0.0226, *p* = 0.9165), respectively.

### 3.3. Relationships between Levels of PPARα and PIP5K1C, PIP2, PI3K (p85/p55) and PTEN in HT-29 Cell Line

The expression of PPARα in HT-29 cells under the same experimental condition was described previously [18]. Expression levels of the antigens of interest obtained with In-Cell ELISA showed several significant correlations. Statistically significant moderate positive correlations were found between PPARα and PIP5K1C (Spearman r = 0.6364, *p* = 0.0290, *n* = 12) as well as between PPARα and PIP2 (Spearman r = 0.6812, *p* = 0.0147, *n* = 12), whereas PPARα and PTEN expression showed a statistically significant moderate negative correlation (Spearman r = −0.6014, *p* = 0.0428, *n* = 12).

**Figure 2:**
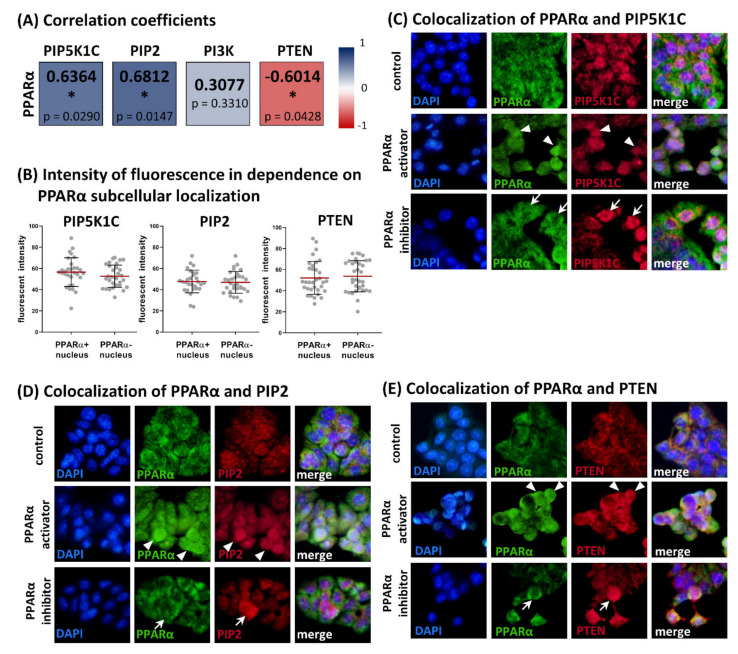
Relationships between levels of PPARα and PIP5K1C, PIP2, PI3K, and PTEN in HT-29 cell line. (**A**) Spearman correlation coefficients (*n* = 12) for expression levels obtained with In-Cell ELISA method. PPARα expression under the same experimental condition was examined in our previous study [18]. Moderate positive correlations were found between PPARα and PIP5K1C and PPARα and PIP2, whereas PPARα and PTEN expression showed moderate negative correlation (statistical significance marked with asterisk, * *p* ≤ 0.05) (**B**) Intensities of PIP5K1C, PIP2, and PTEN immunofluorescent staining for dependence of PPARα subcellular localization. Fluorescent intensities of PIP5K1C, PIP2 and PTEN were measured using ImageJ software. Note that intensities of fluorescence are at the same levels in cells with nucleus positive for PPARα and cells with PPARα negative nucleus (unpaired *t*-test, nonsignificant results, *n* = 30 in each group). Colocalization of PPARα and PIP5K1C (**C**), PIP2 (**D**), and PTEN (**E**). The cells were treated by 150 μM fenofibrate (PPARα activator) and 10 μM GW6471 (PPARα inhibitor), and controls were treated with appropriate amount of DMSO. Staining intensities of PIP5K1C, PIP2, and PTEN were independent of subcellular localization of PPARα. White arrows indicate cells with PPARα negative nuclei together with strong staining of PIP5K1C, PIP2, and PTEN. White arrow heads indicate cells with PPARα positive nuclei together with intensive staining of PIP5K1C, PIP2, and PTEN. All microphotographs are in the same magnification (400×), white line represents 10 μm.

The colocalization of the antigens of interest with PPARα showed no dependence of PIP5K1C, PIP2, and PTEN expression levels (immunostaining intensities) on the subcellular localization of PPARα. We detected strong immunofluorescence of PIP5K1C, PIP2, and PTEN in cells with PPARα positive as well as PPARα negative nuclei (see Figure 2). It suggests that the receptor is not directly involved in transcriptional regulation of the antigens of interest, although the total levels of studied antigens and PPARα showed statistically significant relationships. This observation was confirmed by image analysis. The fluorescent intensities of PIP5K1C, PIP2, and PTEN were measured separately in cells with PPARα positive nuclei and in cells with PPARα negative nuclei. There were comparable levels of immunofluorescence detected between these groups for all three evaluated antigens, with the following *p*-values: 0.2165 for PIP5K1C, 0.7842 for PIP2, and 0.6648 for PTEN (unpaired *t*-test, *n* = 30). For results, see Figure 2.

### 3.4. Comparison of PIP5K1C, PIP2, PI3K (p85α), PTEN and PPARα in Grade 3 Colorectal Carcinomas and Adjacent Normal Tissue Samples

We detected a positive immunostaining for all four studied antigens in all normal colon tissue samples (*n* = 8). We found a statistically significant decrease in the expression of PTEN in tumors (median 1; low staining) in comparison to adjacent normal colon tissue samples (median 2; moderate staining; *p* = 0.0078). Moreover, in normal tissue, PTEN was localized predominantly in the nucleus, whereas in tumor samples, this protein was detected mainly in the cytoplasm. In addition to the epithelium, the connective tissue cells were also positive.

**Figure 3:**
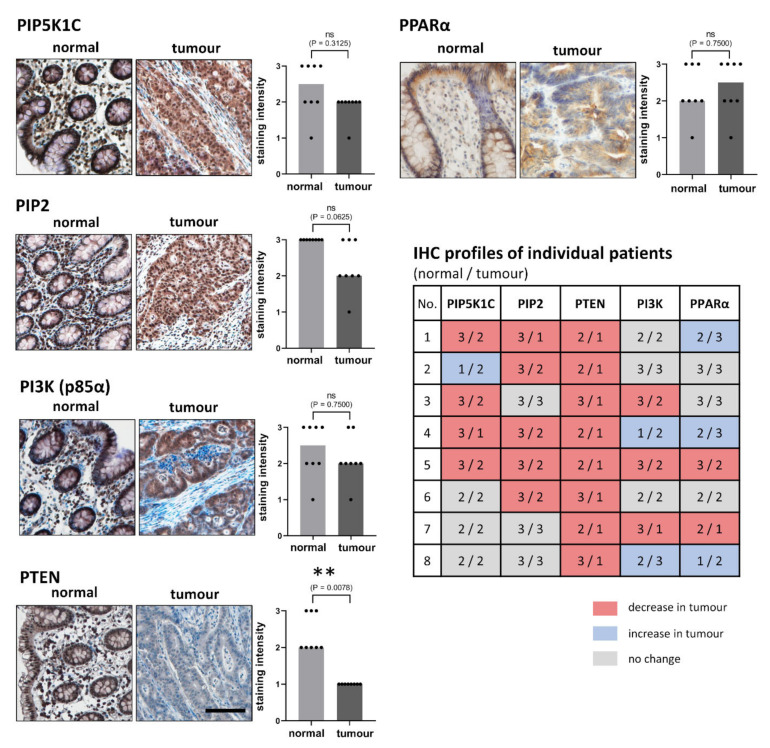
Immunohistochemical staining of PIP5K1C, PIP2, PI3K, PTEN, and PPARα in normal and colorectal carcinoma samples. Representative microphotographs of poorly differentiated (grade 3) tumors and corresponding normal tissue samples were obtained from the same patient (male, 69 years old, no. 5). Magnification 100×, black line represents 100 μm. Graphs show immunohistochemical staining intensities (*n* = 8): columns show medians, dots show individual samples. Immunostaining intensities were evaluated in semiquantitative manner as: 0 = negative staining, 1 = low staining, 2 = moderate staining, 3 = strong staining. Comparison of staining intensities between normal and carcinoma tissues was performed by Wilcox test at level of significance *p* ≤ 0.05. Statistically significant results are marked by (** *p* ≤ 0.01), *p*-values are given directly in graphs. Table represents individual IHC profiles of each patient, i.e., staining intensities for adjacent normal tissue/tumor, patient no. corresponds with their no. in Table 1. Note very similar patterns of changes between the expression of PPARα and PI3K (p85α).

The slight (and nonsignificant) decrease in immunostaining intensities between adjacent normal tissues and tumors was found for PIP5K1C and PI3K. In both cases the median of the immunostaining was 2.5 (moderate/strong staining) for normal tissue, and a median of 2 (moderate staining) was observed for tumors. The PIP2 immunostaining was also decreased in tumor (median 3, strong staining) for normal tissue vs. median 2 (moderate staining) for tumors. PIP5K1C was found in nuclear and cytoplasmic localization in both, carcinomas and normal colon. PIP2 showed mainly nuclear localization in both, carcinomas and colon tissues. For PI3K (p85α), a different subcellular localization between carcinomas and normal tissues was detected. In normal colon tissue, PI3K (p85α) was localized dominantly in the cell nuclei, whereas in carcinomas, it was found in the cytoplasm. The expression of PPARα for a wider set of samples was published in our previous study [18]. Here, in Figure 3, we present only the results for grade 3 carcinomas. There was no significant difference between normal and tumor tissues although there was slight increase in staining intensity in tumors (median 2, moderate staining) for normal tissue vs. median 2.5 (moderate/strong staining for tumors).

Moreover, we compared changes between the tumors and adjacent normal tissue samples for each antigen of interest for each patient to obtain individual immunohistochemical profiles. Contrary to our in vitro study, where expression levels of PPARα revealed relationships for PIP5K1C, PIP2, and PTEN, we surprisingly observed similar trends in changes of expression patterns between PI3K (p85α) and PPARα. The same trend was observed in 6/8 patients.

## 4. Discussion

PPARα is a well-known lipid sensor with various functions in organisms such as energy metabolism, oxidative stress, immune response, and xenobiotic metabolism. Moreover, the PPARα activators fenofibrate and WY-14643 as well as the PPARα inhibitor GW6471 may increase the expression of villin, a marker of intestinal cell differentiation, in vitro, as we described in our previous study [18]. It is known that association of lipid messenger PIP2 with villin enhances its actin bundling function and thus formation of brush borders [19,20,21]. Thus, we focused on the effect of PPARα ligands on lipid messenger PIP2 and enzymes which are involved in its turnover, such as PIP5K1C, PI3K, and PTEN.

In accordance with our previously reported results [18], we showed that fenofibrate, WY-14643, and GW6471 raised levels of PIP2. The same experimental conditions led to an increase in villin expression together with a decrease in cell proliferation [18], suggesting the central role of PIP2 in the response of HT-29 and Caco2 cells to the PPARα ligands tested. Although fenofibrate, WY-14643, and GW6471 increased levels of PIP2, this effect is probably reached by affecting the expression of different enzymes which are involved in PIP2 turnover. The tested PPARα ligands affected mainly the expression of PIP2-producing enzymes PIP5K1C and PTEN. PIP5K1C synthesizes PIP2 by introducing phosphate to phosphatidylinositol-4-phosphate, whereas PTEN creates PIP2 by removing phosphate from PIP3 and thus reverses the action of PI3K [27]. Our results showed that the effect of the tested compounds differed not only between PPARα activators on one side and the PPARα inhibitor on the other side but also between the PPARα activators themselves. The PPARα activators fenofibrate and WY-14643 showed an inverse effect on the expression of PTEN and PIP5K1C. Surprisingly, the effect of the PPARα inhibitor GW6471 stood in between the effects observed for fenofibrate and WY-14643. Although the exact mechanism of action of fenofibrate and WY-14643 is elusive, differences between their binding to PPARα could lead to different biological responses. Fenofibric acid, an active form of fenofibrate, and WY-14643 molecules take different orientations in the ligand-binding domain of PPARα [31]. It was also reported that both WY-14643 and fenofibric acid possess two binding sites [32].

Although we observed a significant relationship between the expression levels of PPARα and PIP5K1C and between PPARα and both PTEN and the PPARα lipid messenger PIP2, the expression of these antigens was independent of the subcellular localization of PPARα. Because nuclear localization of the receptor is the first prerequisite for transcriptional regulation of its targets, our finding suggests that changes in PIP5K1C and PTEN expression are not directly caused by the binding of PPARα to promoter of the target genes. The effect of the PPARα activators and the PPARα inhibitor on levels of proteins/lipids studied was reached at concentrations which decrease cell proliferation to 70–90% of control [18]. It has been reported that these higher concentrations of PPARα activators could also activate PPARγ and/or PPARβ/δ. Fenofibrate and WY-14643 could activate PPARγ with approximately 10-fold selectivity for PPARα. Moreover, the latter could also activate PPARβ/δ with 7-fold selectivity for PPARα [33]. The expression of all three PPAR isoforms in the HT-29 and Caco2 cell lines has been detected previously [34]. It has been reported that, in colorectal carcinoma cells, PPARγ stimulates the expression of PTEN and inhibits PI3K activity [35,36]. Ligand activation of PPARβ/δ had no effect on expression of PTEN or phosphorylation of Akt in the mouse colon [37]. Based on these, one can speculate that the activation of PPARγ could contribute to the effect observed for WY-14643 on PTEN expression but not the effect observed for fenofibrate.

PI3K/Akt/PTEN signaling is one of the important pathways involved in the differentiation of intestinal cells as well as in colorectal carcinomas. However, the role of PI3K/Akt/PTEN signaling in cellular differentiation is unclear, with conflicting reports of promotion or inhibition of the differentiation process [38,39]. Wang et al. [39] showed that inhibition of PI3K or activation of PTEN can increase the expression of brush border enzymes such as sucrase-isomaltase and intestinal alkaline phosphatase in HT-29 and Caco2 cell lines. Additionally, Kim et al. [40] reported that PTEN expression is higher in differentiated cells at the surface epithelium of the murine colon than in undifferentiated ones located in the crypt area. Contradictory results were reported by Laprise et al. [38]. They showed that PI3K activity promotes differentiation of Caco2/15 cells. In their study, inhibition of PI3K decreased the expression of sucrase-isomaltase and villin and reduced cell polarization and brush border formation.

To elucidate the relationship between PPARα and protein/lipid messenger in intestinal cell differentiation, we detected antigens of interest in poorly differentiated colorectal carcinomas and adjacent normal tissues. In accordance with previous studies [41,42], we found a significant decrease in PTEN expression in poorly differentiated carcinomas compared to corresponding non-tumor tissues. However, it was the only significant difference between tumor and normal tissue observed in our set of patient samples. Neither in PIP5K1C nor in the regulatory subunit of PI3K (p85) did immunostaining intensities statistically change. Contrary to this, the recent study conducted by Peng et al. [43] showed that PIP5K1C is highly expressed in colorectal cancer and predicts a poor prognosis. Moreover, it has been reported that colorectal carcinomas are associated with overexpression of both the catalytic (p110α) and regulatory (p85) subunits of PI3K. In particular, mutations in the PI3K catalytic subunit are associated with the gain of function of this enzyme [41,44,45,46,47]. Moreover, comparison of individual IHC profiles obtained for each patient showed that changes in PPARα expression between tumors and adjacent normal tissues resembled those observed for regulatory subunit of PI3K in 6/8 patients. It was an unexpected finding because in vitro experiments with the PPARα activators and inhibitor in colorectal carcinoma cells showed relationship in expression of PPARα with PIP5K1C and PTEN enzymes but not with PI3K. Unfortunately, patients in our study were not mutation-typed for PI3K and maybe this information could shed on light on the discrepancy between the in vitro results and the patient samples. Lastly, it is necessary to bear in mind that, in tumor tissues, PPARα could be affected by various ligands, including fatty acids and pollutants from the environment such as phthalates or pesticides [1], which could induce the expression of different target genes than in our in vitro study.

An interesting finding was a similar change in the subcellular localization of PTEN and PI3K regulatory subunit (p85α) in carcinomas. Whereas in non-tumor tissues the immunostaining was highly detected in the cell nuclei, in carcinomas it diminished from the nuclei and became almost exclusively cytoplasmic. It was reported earlier that loss of nuclear PTEN expression represents a marker of poor clinical outcome in patients with colorectal cancer [48]. Although our in vitro results showed changes in PTEN expression, the administration of fenofibrate and GW6471 did not affect the subcellular localization of this enzyme in HT-29 cells. It is known that p85 has a tumor-suppressor role in the liver [49] and breast cancer [50]. In addition to regulation of p110 activity, p85 also enhances the function of PTEN [51]. Thus, the observed changes of p85α and PTEN subcellular localization observed in poorly differentiated carcinomas may favor carcinogenesis.

Taken together, we showed that the PPARα ligands fenofibrate, WY-14643, and GW6471 increase the level of the lipid messenger PIP2, an important player in the formation of intestinal cell brush border. Surprisingly, each tested PPARα ligand affected different PIP2-metabolizing enzymes, especially levels of PIP5K1C and PTEN. Moreover, we found a positive relationship between the levels of PPARα and PIP2 as well as PPARα and PIP5K1C. In contrast, PPARα was in negative correlation with PTEN. However, the expression of the antigens of interest was independent of the subcellular localization of PPARα, suggesting that PPARα is not directly involved in their regulation. In colorectal carcinoma tissues we found a decrease of PTEN expression which was accompanied by a change in its subcellular localization. However, this change was independent of PPARα expression in tumors. The change of subcellular localization was also observed for the regulatory subunit of PI3K.

## Figures and Tables

**Table 1:** Basic characteristics of patients used in this study. c.—colon.

No.	Sex	Age	Diagnosis	Localization	TNM Staging			Grading
					T	N	M	
1	male	66	adenocarcinoma	c. sigmoideum	T3	N0	M0	G3
2	male	63	adenocarcinoma	c. descendens, rectum	T3	N2b	M0	G3
3	male	54	adenocarcinoma	c. sigmoideum	T2	N0	M0	G3
4	female	70	adenocarcinoma	c. descendens, rectum	T3	N2a	M0	G3
5	male	69	adenocarcinoma	c. sigmoideum	T3	N0	M0	G3
6	female	39	adenocarcinoma	c.sigmoideum	T3	N1a	M0	G3
7	male	77	adenocarcinoma	c. sigmoideum	T3	N0	M0	G3
8	female	50	adenocarcinoma	c. sigmoideum	T3	N2b	M1b	G3

## Data Availability

Data is contained within the article or supplementary material.

## Supplementary Materials

The following are available online at https://www.mdpi.com/article/10.3390/biology11070997/s1, Figure S1: Effect of PPARα activators (fenofibrate and WY-14643) and PPARα inhibitor (GW6471) on cell proliferation and expression of villin. These results were published in our previous study. The experiments were performed under the same experimental conditions as in current study. Effect of PPARα ligands on undifferentiated (A), sodium butirate-differentiated (B) HT-29 cells as well as in undifferentiated (C) and spontaneously differentiated (D) Caco2 cells. Relative cell proliferation measured by WST-1 assay. Results are shown as mean ± SD (n = 9). Relative expression of villin in comparison to control was measured by In-Cell ELISA. Results are shown as mean ± SD (n = 6). Red dotted line represents control cells: DMSO treated undifferentiated or differentiated cells (100%). Note the increase in villin expression in both used cell lines after PPARα treatment in concentrations decreasing cell proliferation. Statistically significant results (one sample *t*-test) in comparison to control cells are marked by * *p* ≤ 0.05, ** *p* ≤ 0.01, *** *p* ≤ 0.001, **** *p* ≤ 0.0001.
