# Supplementary material for: Lipid Messenger Phosphatidylinositol-4,5-Bisphosphate Is Increased by Both PPARα Activators and Inhibitors: Relevance for Intestinal Cell Differentiation
Source: Biology (Basel). 2022 Jun 30;11(7):997. doi: 10.3390/biology11070997 (PMC9312331; doi:10.3390/biology11070997)
Supplement: Supplementary file 1 [file biology-11-00997-s001.zip › biology-1745425-supplementary.pdf]

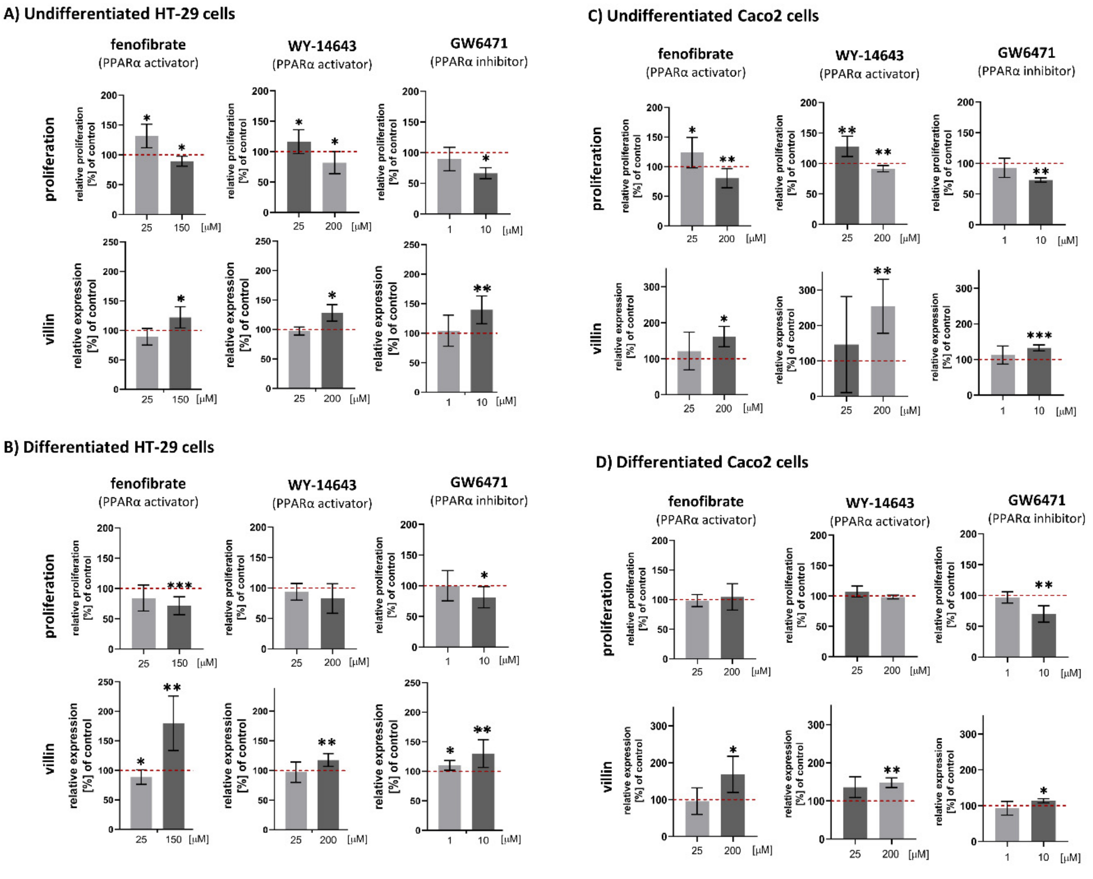

Figure S1. Effect of PPARα activators (fenofibrate and WY-14643) and PPARα inhibitor (GW6471) on cell proliferation and expression of villin. These results were published in our previous study. The experiments were performed under the same experimental conditions as in current study. Effect of PPARα ligands on undifferentiated (A), sodium butirate-differentiated (B) HT-29 cells as well as in undifferentiated (C) and spontaneously differentiated (D) Caco2 cells. Relative cell proliferation measured by WST-1 assay. Results are shown as mean ± SD (n = 9). Relative expression of villin in comparison to control was measured by In-Cell ELISA. Results are shown as mean ± SD (n = 6). Red dotted line represents control cells: DMSO treated undifferentiated or differentiated cells (100%). Note the increase in villin expression in both used cell lines after PPARα treatment in concentrations decreasing cell proliferation. Statistically significant results (one sample t-test) in comparison to control cells are marked by * $p \leq 0.05$, ** $p \leq 0.01$, *** $p \leq 0.001$, **** $p \leq 0.0001$.
